# Early Afterdepolarizations with Growing Amplitudes via Delayed Subcritical Hopf Bifurcations and Unstable Manifolds of Saddle Foci in Cardiac Action Potential Dynamics

**DOI:** 10.1371/journal.pone.0151178

**Published:** 2016-03-15

**Authors:** Philipp Kügler

**Affiliations:** 1 Institute of Applied Mathematics and Statistics, University of Hohenheim, Schloss 1, 70599 Stuttgart, Germany; 2 Research Group Mathematical Methods in Molecular and Systems Biology, Radon Institute for Computational and Applied Mathematics, Altenbergerstrasse 69, 4040 Linz, Austria; University of Minnesota, UNITED STATES

## Abstract

Early afterdepolarizations (EADs) are pathological oscillations in cardiac action potentials during the repolarization phase and may be caused by drug side effects, ion channel disease or oxidative stress. The most widely observed EAD pattern is characterized by oscillations with growing amplitudes. So far, its occurence has been explained in terms of a supercritical Hopf bifurcation in the fast subsystem of the action potential dynamics from which stable limit cycles with growing amplitudes emerge. The novel contribution of this article is the introduction of two alternative explanations of EAD genesis with growing amplitudes that do not involve stable limit cycles in fast subsystems. In particular, we demonstrate that EAD patterns with growing amplitudes may alternatively arise due to a delayed subcritical Hopf bifurcation or an unstable manifold of a saddle focus fixed point in the full fast-slow system modelling the action potential. Our work extends the list of possible dynamical EAD mechanisms and may contribute to a classification of drug effects in preclinical cardiotoxicity testing.

## Introduction

**Fig 1:**
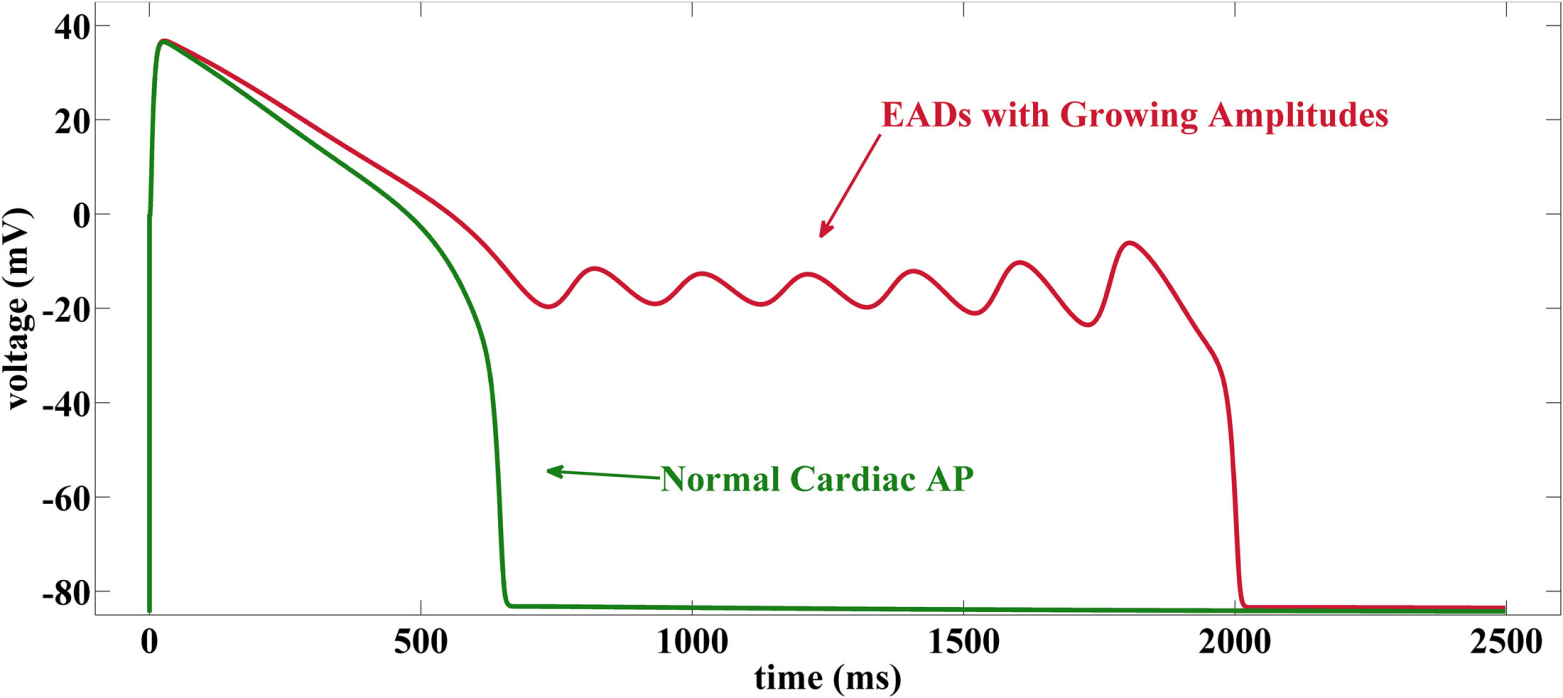
Cardiac AP and Distortion by EADs. Green curve shows simulation of cardiac action potential with depolarization due to superthreshold stimulation and normal repolarization back to resting potential. Red curve shows an AP distorted by early afterdepolarizations with growing amplitudes.

The term action potential (AP) refers to the characteristic membrane voltage response of excitable cells such as cardiomyocytes to a superthreshold electric stimulus, see Fig 1. Cardiac APs are regulated by a subtle interplay of various ion channels [1] that control the in- and outflow of ions across the membrane. If this interplay is perturbed by pharmaceutical compounds [2], oxidative stress [3] or cardiac disease [4], the AP gets impaired and early afterdepolarizations (EADs) may arise. EADs are pathological voltage oscillations during the AP repolarization (or plateau) phase, see Fig 1, that may synchronize and trigger potentially lethal ventricular fibrillation [5].

At the cellular level, cardiac APs are mathematically described by means of coupled systems of nonlinear ODEs that consider the cellular membrane as an electrical circuit consisting of a capacitative current in parallel with several transmembrane ionic currents. Therein, the voltage equation

$$\begin{array}{c} C\frac{dV}{dt}=-\sum_{ion}I_{ion} \end{array}$$

is complemented by additional ODEs for channel gating variables that describe the voltage dependent activation and deactivation of the ionic currents. Modern cardiac AP models for animal [6], human adult [7] and human induced pluripotent stem cell derived [8] cardiomyocytes comprise dozens of state variables and hundreds of model parameters.

Using AP models of lower dimension, cardiac arrhythmias have been associated with bifurcations [9, 10] in the nonlinear AP dynamics. In particular, the on- and offset of EADs have been linked in [11, 12] with supercritical Hopf and saddle-homoclinic bifurcations in the fast subsystem of a four dimensional AP model. This bifurcation scenario features stable limit cycles with growing amplitudes and currently is considered [13] to be necessary for the occurence of EADs with growing amplitudes (the most widely observed EAD pattern in experiments).

In this paper we demonstrate that stable limit cycles in the fast subsystem of cardiac AP models are not the only possible explanation for EAD patterns with growing amplitudes. In particular we illustrate that this EAD pattern may also result from a delayed subcritical Hopf bifurcation or an unstable manifold of a saddle focus fixed point in the full AP dynamics- two scenerios that do not involve stable limit cycles in the fast subsystem. That way, our manuscript offers two novel hypotheses on the generation of EADs with growing amplitudes.

## Materials and Methods

### Cardiac Action Potential Models

#### Four Dimensional AP Model

The current bifurcation hypothesis on the generation of EADs with growing amplitudes featured in [9, 10, 12, 13] was introduced in [11]. Therein, the authors used the AP model

$$\begin{array}{ccc} C\frac{dV}{dt} & = & -G_{Ca}df\left(V-E_{Ca}\right)-G_{K}x\bar{x}\left(V\right)\left(V-E_{K}\right)-I_{0}\left(V\right),\\ \frac{dd}{dt} & = & \frac{d_{\infty }\left(V\right)-d}{\alpha \tau_{d}\left(V\right)},\\ \frac{df}{dt} & = & \frac{f_{\infty }\left(V\right)-f}{\beta \tau_{f}\left(V\right)},\\ \frac{dx}{dt} & = & \frac{x_{\infty }\left(V\right)-x}{\gamma \tau_{x}\left(V\right)}, \end{array}$$ (1)

which is a reduced version of the Luo-Rudy model [14] for mammalian ventricular cells. This model includes the inward calcium current

$$\begin{array}{c} I_{Ca}=G_{Ca}df\left(V-E_{Ca}\right) \end{array}$$

with the calcium channel conductance *G*_*Ca*_ and the dynamic activation and inactivation variables *d* and *f* as well as the outward potassium current

$$\begin{array}{c} I_{K}=G_{K}x\bar{x}\left(V\right)\left(V-E_{K}\right) \end{array}$$

with the potassium channel conductance *G*_*K*_ and the dynamic activation variable *x*. The voltage dependent functions of the model include the inactivation variable $\bar{x}$, the background current *I*_0_ as well as the relaxation variables *τ*_*d*_, *τ*_*f*_, *τ*_*x*_ and the steady states *d*_∞_, *f*_∞_, *x*_∞_ of channel gating.

For biophysical reasons one may argue that the activation of the potassium current is a much slower process than the activation and deactivation of the calcium current [1]. This motivates the consideration of Eq (1) as a (3, 1) fast-slow system with the fast variables *V*, *d*, *f* and the slow variable *x*. Then, the associated fast subsystem is given by

$$\begin{array}{ccc} C\frac{dV}{dt} & = & -G_{Ca}df\left(V-E_{Ca}\right)-G_{K}x\bar{x}\left(V\right)\left(V-E_{K}\right)-I_{0}\left(V\right),\\ \frac{dd}{dt} & = & \frac{d_{\infty }\left(V\right)-d}{\tau_{d}\left(V\right)},\\ \frac{df}{dt} & = & \frac{f_{\infty }\left(V\right)-f}{\tau_{f}\left(V\right)}. \end{array}$$ (2)

#### Three Dimensional AP Model

An even simpler cardiac AP model is

$$\begin{array}{ccc} C\frac{dV}{dt} & = & -G_{Ca}d_{\infty }\left(V\right)f\left(V-E_{Ca}\right)-G_{K}x\left(V-E_{K}\right),\\ \frac{df}{dt} & = & \frac{f_{\infty }\left(V\right)-f}{\tau_{f}},\\ \frac{dx}{dt} & = & \frac{x_{\infty }\left(V\right)-x}{\tau_{x}}, \end{array}$$ (3)

which was introduced in [15] for the analysis of chaotic AP dynamics. The model (3) was also used in [16] to simulate the transient impact of *β*-adrenergic ion channel stimulators. In comparison with Eq (1) the fast variable *d* is replaced by its steady state *d*_∞_(*V*) and the relaxation variables *τ*_*f*_ and *τ*_*x*_ are considered to be constant. Further simplifications are given by $\bar{x}\left(V\right)=1$ and *I*_0_(*V*) = 0.

Viewing Eq (3) as a (2, 1) fast-slow system with fast variables *V*, *f* and slow variable *x* yields the fast subsystem

$$\begin{array}{ccc} C\frac{dV}{dt} & = & -G_{Ca}d_{\infty }\left(V\right)f\left(V-E_{Ca}\right)-G_{K}x\left(V-E_{K}\right),\\ \frac{df}{dt} & = & \frac{f_{\infty }\left(V\right)-f}{\tau_{f}}. \end{array}$$ (4)

### Simulation of Action Potential Models

For the numerical simulation of the action potential models (1) and (3) we used the MATLAB [17] solver ode15s for stiff ODE systems. Tables 1 and 2 give the respective model parameter values and initial conditions used.

**Table 1:** Parameters and initial conditions for the four dimensional AP model (1).

Parameter	A	B	C
*α*	0.1	0.1	0.1
*β*	1.1	1.1	1.1
*γ*	10	10	10
*G*_*Ca*_	0.15	0.195	0.1275
*G*_*K*_	0.282	0.282	0.282
Initial Condition	A	B	C
*V*_0_	0	0	0
*d*_0_	0.0032	0.0034	0.0032
*f*_0_	0.9999	0.9983	0.9918
*x*_0_	0.2161	0.3637	0.192

Parameter values of column A are taken from [11]. Model parameters not listed are the same as in [11]. By *V*_0_ = 0, the initial conditions mimic the effect of a stimulating current pulse.

**Table 2:** Parameters and initial conditions for the three dimensional AP model (3).

Parameter	A	B	C	D
*τ*_*f*_	80	80	18	18
*τ*_*x*_	300	300	100	100
*G*_*Ca*_	0.025	0.025	0.025	0.025
*G*_*K*_	0.04	0.035	0.04	0.0393
Initial Condition	A	B	C	D
*V*_0_	0	0	0	0
*f*_0_	0.9989	0.9990	0.9986	0.9984
*x*_0_	0.0151	0.0491	0.0145	0.0054

Parameter values of column A are taken from [15], parameter values of column C are taken from [16]. Model parameters not listed are the same as in [15]. By *V*_0_ = 0, the initial conditions mimic the effect of a stimulating current pulse.

### Bifurcation Analysis of Fast AP Subsystems

For studying the genesis of EADs in the full action potential models (1) and (3), we performed a numerical bifurcation analysis of the corresponding fast subsystems Eqs (2) and (4) with *x* as continuation parameter using the software package Matcont [18]. The model parameter values used are given Tables 1 and 2.

## Results

### EADs with Growing Amplitudes via Stable Limit Cycles in the Fast AP Subsystem

**Fig 2:**
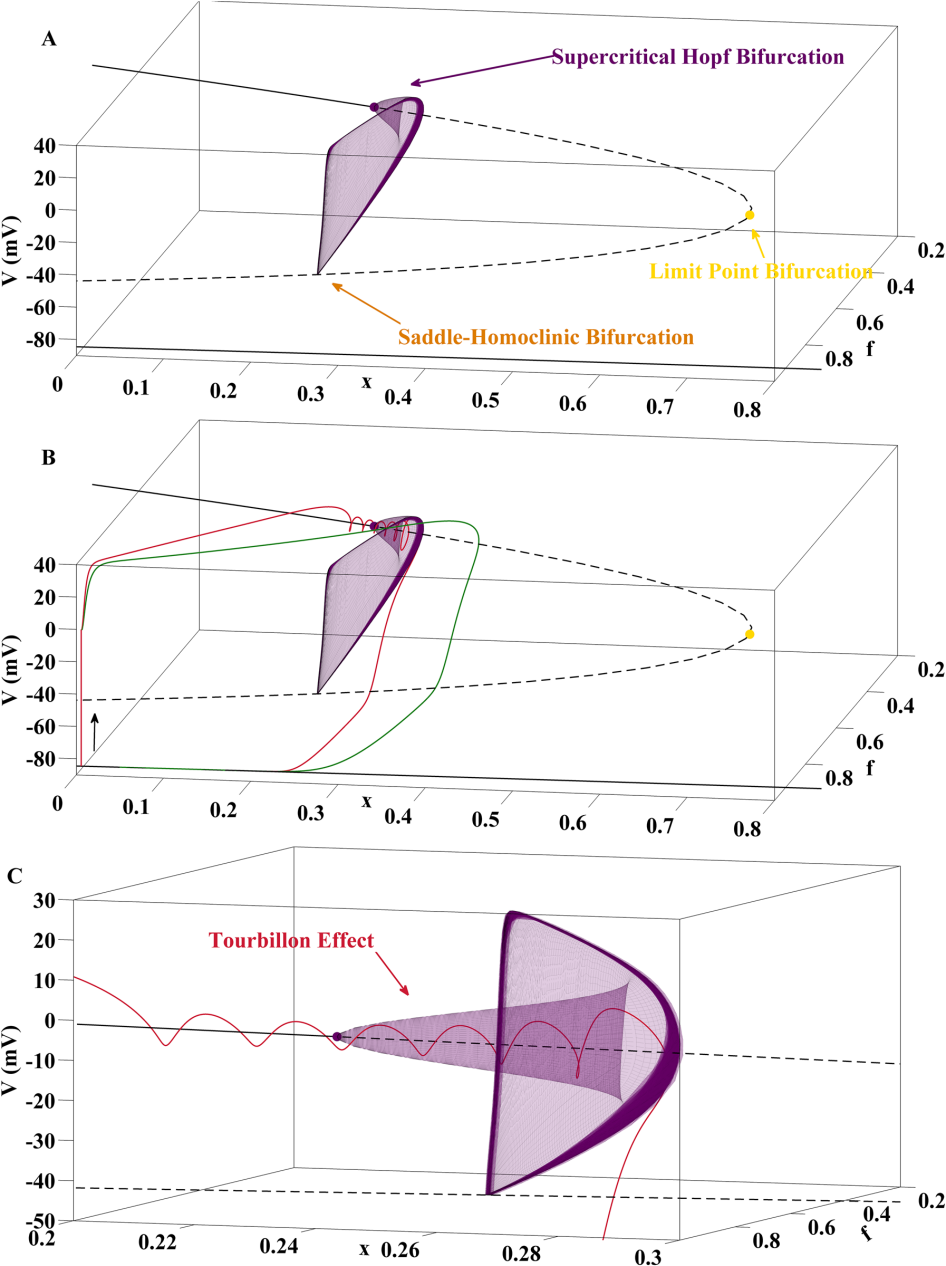
EADs with Growing Amplitudes via Stable Limit Cycles in the AP Fast Subsystem. (A) Bifurcation diagram for the fast subsystem Eq (2) with parameter column A of Table 1 and *x* as continuation parameter. Black solid and dashed curves represent stable and unstable fixed points of Eq (2). At the supercritical Hopf bifurcation the stable focus-nodes turn into unstable fixed points of the saddle-focus type. Furthermore, a branch of stable limit cycles arises which terminates at a saddle-homoclinic bifurcation. At the limit point bifurcation the saddle-focus branch collides with a branch of unstable fixed points of the saddle type. The lower branch of fixed points is formed by stable nodes. (B) Projection of two trajectories of the full system Eq (1) onto the (V,f,x)-space, obtained with parameter column A of Table 1 for the red curve and the substitution *γ* = 4 for the green curve. If the solution curve of Eq (1) passes by the basins of attraction of the fast subsystem, no EADs occur, see green line. EADs with growing amplitudes are generated if the trajectory passes through the supercritical Hopf point of Eq (2) before being pulled towards stable limit cycles with increasing amplitudes, see red line. From the perspective of multiple time scales theory, the situation corresponds to a Tourbillon effect. (C) Zoom into the neighborhood of the supercritical Hopf bifurcation with passage of the EAD carrying trajectory.

First, we review the current bifurcation hypothesis on EAD genesis advertized in [9–13] and put it in context to the phenomenon of delayed Hopf bifurcations [19] in dynamical systems with multiple time scales [20]. The bifurcation analysis [11] of Eq (2) with *x* as continuation parameter reveals that Eq (2) may possess a supercritical Hopf bifurcation at the upper branch of fixed points, see Fig 2A. From that Hopf point a branch of stable limit cycles emerges that subsequently terminates at a saddle-homoclinic bifurcation [21, 22], i.e., at an orbit that is homoclinic to one of the saddle points along the middle branch of fixed points.

The bifurcation scenario illustrated in Fig 2A explains the generation of EADs with growing amplitudes as follows. If the trajectory of the full system Eq (1) is driven into the basin of attraction of the the branch of stable focus-nodes of the fast subsystem Eq (2), a spiral movement is triggered. As the solution curve of Eq (1) progresses along the branch of fixed point towards the supercritical Hopf bifurcation, the amplitudes decrease due to the negative real part of the complex eigenvalues. After the Hopf bifurcation the oscillations start to grow as the trajectory of Eq (1) is attracted towards the stable limit cycles of Eq (2) until the latter are terminated at the saddle-homoclinic bifurcation. The analysis of [11] has been influenced by [23, 24] in which the combination of a supercritical Hopf bifurcation with a saddle-homoclinic bifurcation has been identified as a mechanism that underlies bursting oscillations in neuronal models.

Fig 2B illustrates this EAD generating mechanism by a projection of the solution curves of Fig 1 onto the (*V*, *f*, *x*)-space. If the trajectory passes by the basin of attractions of the fast subsystem Eq (2), no EADs occur such that a normal action potential is generated. The difference between the two solution curves results from different speeds of the *x*-activation (*γ* = 10 vs. *γ* = 4) which, however, does not impact the fast subsystem Eq (2) and hence the location of its fixed points and bifurcation points.

In the theory of multiple time scale dynamics [20], a Hopf bifurcation in a fast subsystem of a fast-slow system in which a slow variable acts as Hopf bifurcation parameter is called a delayed Hopf bifurcation. The term delay accomodates the fact that the solution curve of the full system remains close to the repelling branch of unstable fixed points for a substantial time after the Hopf bifurcation [19]. From that perspective the scenario illustrated in Fig 2C can be referred to as a delayed supercritical Hopf bifurcation with Tourbillon effect since the small scale oscillations in vicinity of the Hopf point are visible.

In our study, we found two additional patterns of EAD generation with growing amplitudes that can be associated with the bifurcation constellation displayed in Fig 1A. In the one case (generated, e.g., with parameter column B in Table 1), the trajectory passes through the supercritical Hopf point with a pronounced delay effect. In the other cases (generated, e.g., with parameter column C in Table 1), the trajectory does not pass through the supercritical Hopf point but is directly attracted towards the stable limit cycles from outside. Details are given in S1 Appendix.

The common basis of the three cases of EAD generation with growing amplitudes mentioned so far is the existence of stable limit cycles with growing amplitudes in the fast subsystem of the AP model. Our subsequent analysis reveals that at least two alternative dynamic mechanisms for the generation of EADs with growing amplitudes exist that both do not involve stable limit cycles in the fast AP subsystem. In the two novel hypotheses to be introduced in the following, EADs with growing amplitudes are either generated via a delayed subcritical Hopf bifurcation or the unstable manifold of a saddle focus in the AP system.

### EADs with Growing Amplitudes via a Delayed Subcritical Hopf Bifurcation in the AP System

This section illustrates how EAD dynamics with growing amplitudes alternatively may occur via a delayed subcritical Hopf bifurcation in the AP system, i.e., via passage of the solution curve through a subcritical Hopf bifurcation in the fast AP subsystem.

**Fig 3:**
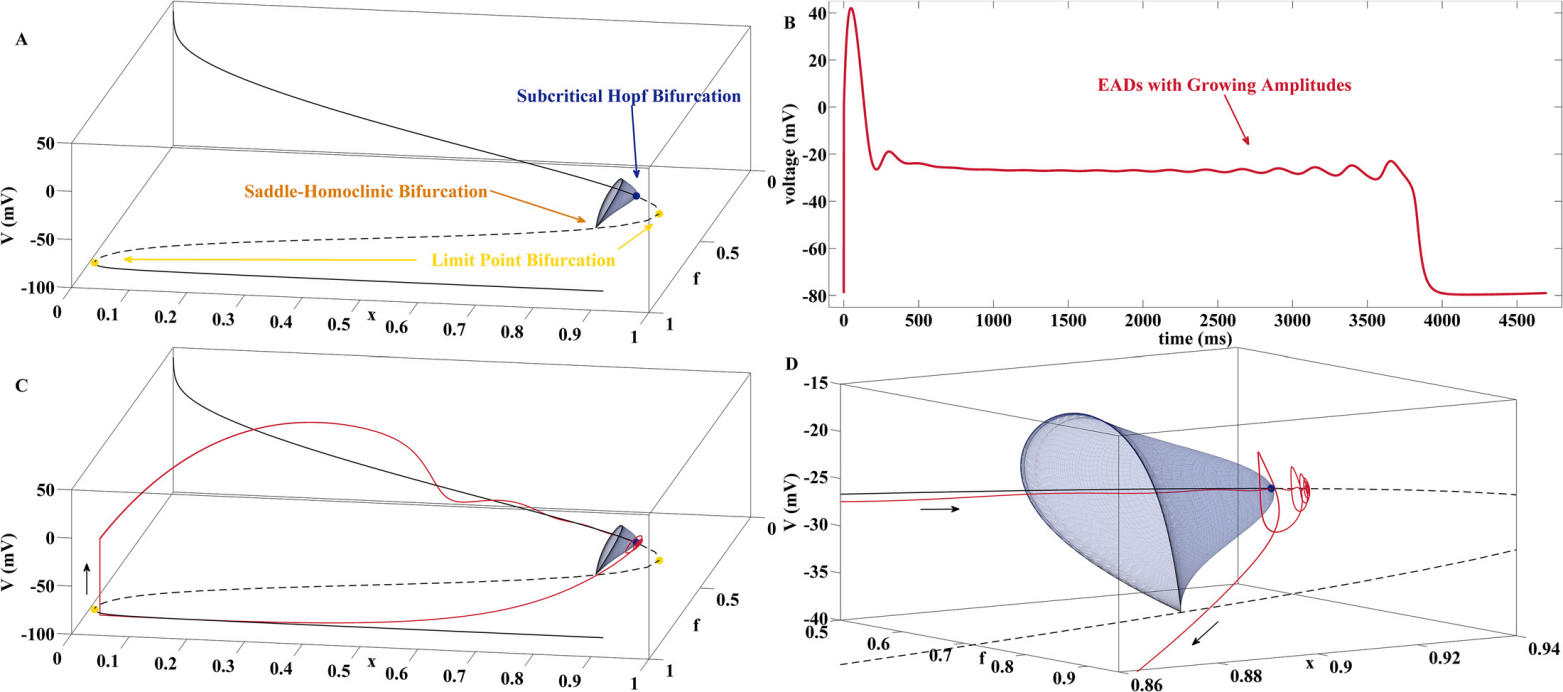
EADs with Growing Amplitudes via a Delayed Subcritical Hopf Bifurcation. (A) Bifurcation diagram for the fast subsystem Eq (4) with *x* as continuation parameter and parameter column B of Table 2. Black solid and dashed curves represent stable and unstable fixed points of Eq (4). At the subcritical Hopf bifurcation the stable foci turn into unstable foci. Furthermore, a branch of unstable limit cycles arises which terminates at a saddle-homoclinic bifurcation. At the limit point bifurcation the unstable focus branch collides with a branch of unstable fixed points of the saddle type. At the second limit point bifurcation, the saddle branch collides with the lower branch of stable nodes. (B) Solution of Eq (3) with parameter column B of Table 2 that carries EADs with growing amplitudes caused by the delayed subcritical Hopf bifurcation. (C) Projection of the trajectory onto the (V,f,x)-space. The spiraling movement persists after the subcritical Hopf point for some time during which the amplitudes grow exponentially. (D) Zoom into the neighborhood of the subcritical Hopf bifurcation with passage of the EAD carrying trajectory.

A bifurcation analysis of the fast subsystem Eq (4) with *x* as continuation parameter and the model parameter values from column B of Table 2 yields the bifurcation diagram displayed in Fig 3A. As in the scenario displayed in Fig 2A, the upper branch of stable fixed points terminates at a Hopf bifurcation, but this time the latter is of the subcritical type. In particular, the limit cycles, that emerge from the Hopf point and continue in opposite direction until their distruction at a saddle-homoclinic bifurcation, now are unstable. Still, this bifurcation constellation admits the emergence of EADs with growing amplitudes, see Fig 3B for a corresponding solution curve of the full system Eq (3) and Fig 3C for its projection onto the bifurcation diagram. First, the trajectory spirals around the branch of stable foci of Eq (4). After passage through the subcritical Hopf point the trajectory is subject to a delay effect which for some time allows the continuation of the spirals but with increasing amplitudes due to a now positive real part of the pair of complex eigenvalues. Finally, the trajectory is repelled and driven towards the lower branch of stable nodes. Note that the saddle-homoclinic bifurcation is no longer involved in the termination of the EADs which is another significant difference to the cases illustrated in Fig 2 and S1 Appendix.

The EAD mechanism shown in Fig 3 can be understood by studying the (2, 1)-fast-slow system

$$\begin{array}{ccc} \frac{dy_{1}}{dt} & = & xy_{1}-y_{2}+y_{1}{\left(y_{1}^{2}+y_{2}^{2}\right)}^{2},\\ \frac{dy_{2}}{dt} & = & y_{1}+xy_{2}+y_{2}{\left(y_{1}^{2}+y_{2}^{2}\right)}^{2},\\ \frac{dx}{dt} & = & \varepsilon , \end{array}$$ (5)

where the fast subsystem obtained with *ε* = 0 corresponds to the normal form of a subcritical Hopf bifurcation [22] at the origin (*y*_1_, *y*_2_, *x*) = (0, 0, 0). The linearization of the fast subsystem around (0, 0) yields

$$\begin{array}{ccc} \frac{d{\tilde{y}}_{1}}{dt} & = & x{\tilde{y}}_{1}-{\tilde{y}}_{2},\\ \frac{d{\tilde{y}}_{2}}{dt} & = & {\tilde{y}}_{1}+x{\tilde{y}}_{2},\\ \frac{dx}{dt} & = & \varepsilon \end{array}$$

with

$$\begin{array}{ccc} {\tilde{y}}_{1}\left(t\right) & = & y_{1,0}e^{xt}\cos\left(t\right)-y_{2,0}e^{xt}\sin\left(t\right),\\ {\tilde{y}}_{2}\left(t\right) & = & y_{1,0}e^{xt}\sin\left(t\right)+y_{2,0}e^{xt}\cos\left(t\right),\\ x(t) & = & \varepsilon t+x_{0}. \end{array}$$

**Fig 4:**
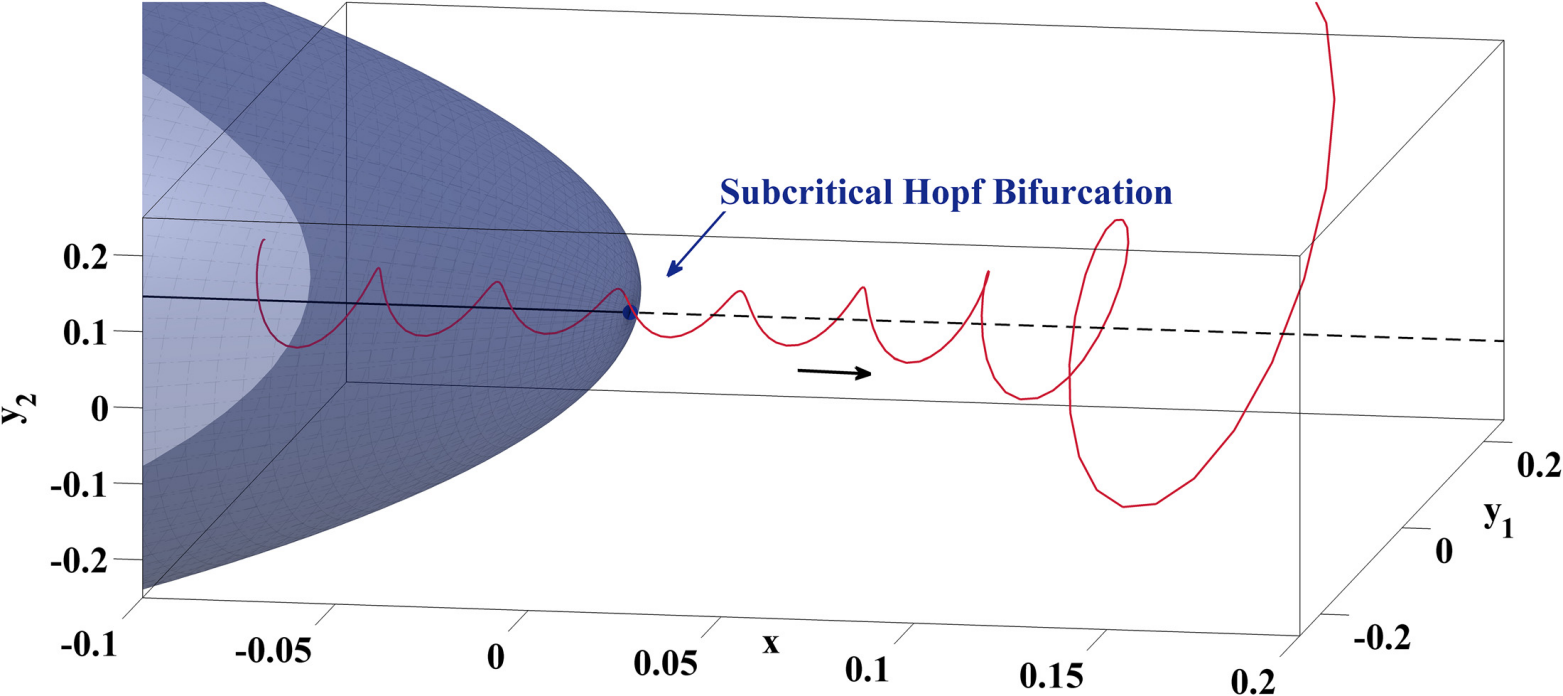
Emergence of Oscillations with Growing Amplitudes due to a Delay Effect Associated with a Subcritical Hopf Bifurcation. Before the trajectory of Eq (5) is repelled from the unstable fixed points of the fast subsystem, it spirals around them with growing amplitudes. Visibility of this effect depends on initial conditions as well as speed of passage and is given, e.g., with (*y*_1,0_, *y*_2,0_, *x*) = (0.05,0.05,−0.1) and *ε* = 0.005.

Hence, for the neighborhood with *x* > 0 the amplitudes of the oscillatory solution always grow exponentially before the trajectory of Eq (5) is repelled from the branch of unstable fixed points. It depends on the initial conditions and the speed *ε* at which the continuation parameter *x* crosses the subcritical Hopf point, if those oscillations become actually visible, see Fig 4 for an illustration.

### EADs with Growing Amplitudes via the Unstable Manifold of a Saddle Focus in the AP System

In this section, we finally demonstrate that EADs with growing amplitude may also occur without the existence of delayed Hopf bifurcations in the AP system. Then, the EAD generating mechanism rather is the unstable manifold a saddle-focus fixed point (V*, f*, x*) of the AP system that coincides with the location of the limit point bifurcation of the fast AP subsystem.

**Fig 5:**
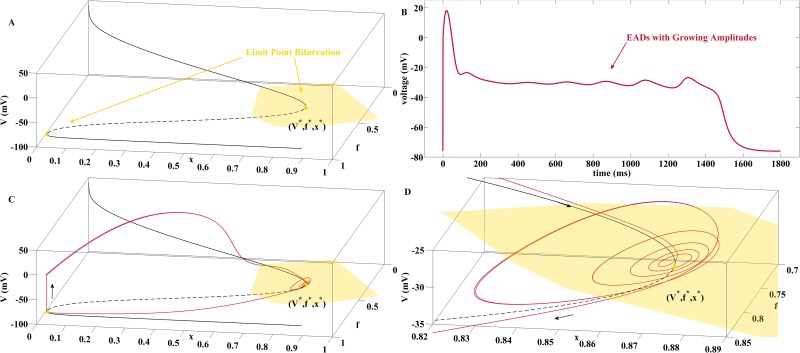
EADs with Growing Amplitudes via the Unstable Manifold of a Saddle Focus. (A) Bifurcation diagram for the fast subsystem Eq (4) with *x* as continuation parameter and model parameters from column D of Table 2. Black solid and dashed curves represent stable and unstable fixed points of Eq (4). The upper branch consists of stable foci which—as opposed to Fig 3A—only terminates at a limit point bifurcation with coordinates (V*, f*, x*) where it turns into a branch of saddles. At the second limit point bifurcation, the saddle branch collides with the lower branch of stable nodes. (B) Solution of Eq (3) with parameter column D of Table 2 that carries EADs with growing amplitudes. They are caused by a saddle focus fixed point of the full system Eq (3) that coincides with the location (V*, f*, x*) of the limit point bifurcation of the fast subsystem Eq (4). (C) Projection of the trajectory onto the (V,f,x)-space. The spiraling movement of the trajectory is caused by the unstable manifold (yellow surface) spanned by the complex conjugate eigenvectors of the Jacobian *J* at (V*, f*, x*). (D) Zoom into the neighborhood of (V*, f*, x*).

A bifurcation analysis of the fast subsystem Eq (4) with *x* as continuation parameter and the model parameter values from column D of Table 2 yields the bifurcation diagram displayed in Fig 5A. The upper branch of fixed points only consists of stable foci that terminate at a limit point bifurcation of Eq (4) such that no Hopf bifurcation exists. Still, the full system Eq (3) may admit EAD trajectories with growing amplitudes, see Fig 5B for a corresponding solution curve and Fig 5C for its projection onto the bifurcation diagram. First, the trajectory spirals around the branch of stable foci of Eq (4) and approaches with decreasing amplitudes the limit point bifurcation located at (V*, f*, x*).

Further analysis of the full system Eq (3) reveals that (V*, f*, x*) coincides with a saddle focus fixed point of the latter that is associated with a one-dimensional stable and a two-dimensional unstable manifold. This manifold is spanned by the pair of complex conjugate eigenvectors of the Jacobian *J* of Eq (3) evaluated at (V*, f*, x*), illustrated as yellow surface in Fig 5C. In vicinity of (V*, f*, x*) the trajectory of Eq (3) is diverted into the unstable manifold which triggers oscillations with growing amplitudes. Finally, the trajectory is repelled resulting in another turn around the stable foci of Eq (4) before crossing the separatrix and being attracted by the lower branch of stable nodes.

**Fig 6:**
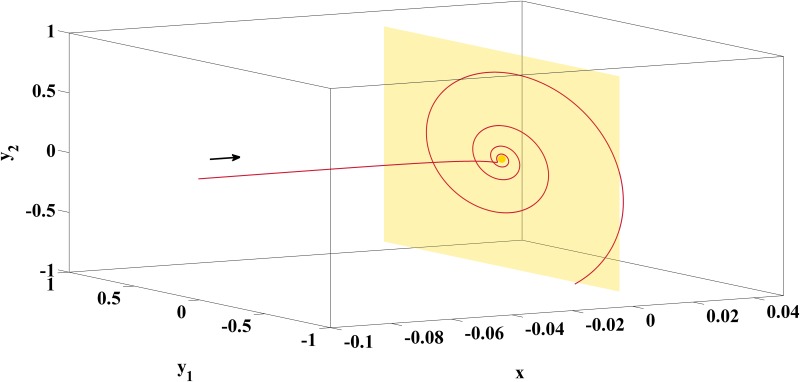
Emergence of Oscillations with Growing Amplitudes due to the Unstable Manifold of a Saddle Focus. The spiraling part of the trajectory lies on the unstable manifold (yellow surface) which is spanned by the pair of complex conjugate eigenvectors of the system matrix, illustrated by means of the linear system Eq (6) with initial conditions (*y*_1,0_, *y*_2,0_, *x*_0_) = (0.01, 0.01, −0.1).

The EAD mechanism illustrated in Fig 5 can be understood by studying the linear system

$$\frac{d}{dt}\left(\begin{array}{c} y_{1}\\ y_{2}\\ x \end{array}\right)=\left(\begin{array}{ccc} 0.0043 & -0.0285 & 0\\ 0.0285 & 0.0043 & 0\\ 0 & 0 & -0.0304 \end{array}\right)\cdot \left(\begin{array}{c} y_{1}\\ y_{2}\\ x \end{array}\right)$$ (6)

which has a saddle focus at (0,0,0). The eigenvalues of the system matrix of Eq (6) are *λ*_1,2_ = 0.0043 ± 0.0285⋅*i*, *λ*_3_ = −0.0304 and coincide with those of *J*(V*, f*, x*) for Eq (3) with parameter column D of Table 2. The stable manifold is given by the *x*-axis while the unstable manifold coincides with the (*y*_1_, *y*_2_)-plane. Fig 6 displays the behaviour of the trajectory *z*(*t*) = *e*^*At*^ *z*_0_ for *z*_0_ = (*y*_1,0_, *y*_2,0_, *x*_0_) = (0.01, 0.01, −0.1) and illustrates how oscillations with growing amplitudes arise on the unstable manifold of the saddle focus.

## Discussion

**Fig 7:**
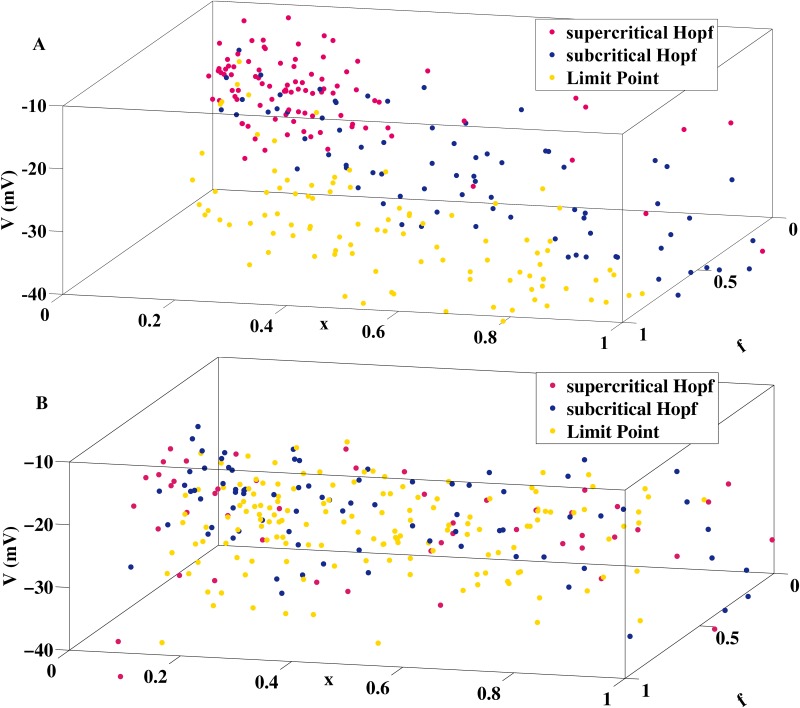
Ubiquity of EAD Generating Mechanisms. The dynamic repertoire of both AP models (3) and (1) comprises all three mechanisms for the generation of EADs with growing amplitudes. (A) The figure displays the location of the corresponding bifurcation points (supercritical Hopf, subcritical Hopf and limit point) of Eq (4) in the (*V*, *f*, *x*)-space obtained by random variation of parameter column A of Table 1. (B) Distribution of EAD generating bifurcation points of Eq (2) as a result of a random variation of parameter column A of Table 2.

EADs are pathological voltage oscillations during the repolarization phase of cardiac APs. The most widely observed EAD pattern is characterized by growing amplitudes and has been previously linked [11–13] with a supercritical Hopf bifurcation in the fast subsystem of AP models. Using the parsimonious AP model (3) we in this paper have associated EADs with growing amplitudes with two alternative dynamical mechanisms, namely a delayed subcritical Hopf bifurcation and an unstable manifold of a saddle focus fixed point in the AP system. As illustrated by Fig 7, the identified EAD mechanisms are not specific to the AP model used for their explanation. Random sampling of the parameter space using multivariate normal distributions (with the parameter columns A of Tables 1 and 2 taken as mean vectors) shows that both models (3) and (1) feature all the bifurcation scenarios outlined in the paper (delayed supercritical Hopf bifurcation, delayed subcritical Hopf bifurcation and saddle focus fixed point in the full AP system). The bifurcation analysis of the more complex state-of-the-art AP models for animal [6], human adult [7] and human induced pluripotent stem cell derived [8] is the subject of future work in which we expect to find an even richer repertoire of possible EAD mechanisms. Furthermore, as the EAD hypothesis of [11] based on the combination of a supercritical Hopf bifurcation with a saddle-homoclinic bifurcation has a counterpart in the context of bursting oscillations in neuronal models [23, 24], the two alternative EAD hypotheses introduced in this paper might also motivate novel contributions in the field of mathematical neuroscience.

Given several in-silico dynamical mechanisms for EAD generation, another future challenge is to determine if and how they can be validated experimentally. One idea is to associate the frequency spectrum of recorded voltage traces that carry EAD patterns with the different periodicities of the stable and unstable oscillatory orbits of the mathematical models. If successful, this might lead to a first bifurcation theory based classification of experimentally obtained EADs.

Knowledge of the actual dynamic EAD mechanism might also serve as a basis for the development of antiarrhythmic drugs for the prevention of cardiac arrhythmias. Given an unfavourable bifurcation scenario one then needs to identify model components that both can be targeted by drugs and, if correspondingly altered, reduce or even eliminate the risk of EAD generation. One possible mathematical approach is to use inverse bifurcation analysis [25, 26] which, however, needs to be extended to, e.g., subcritical Hopf bifurcations or limit point bifurcations that coincide with saddle focus fixed points.

Finally, an understanding of the dynamic EAD mechanisms might also contribute to an improvement of preclinical drug cardiotoxicity testing. While cardiac AP models are currently used to simulate the impact of drugs on the AP trajectory [27], it might be more illuminative to directly study the drug impact on the bifurcation properties. Then, the latter may be used to define novel classifications of the proarrhythmic risk of candidate drugs.

For the sake of completeness we mention that cardiac AP models may also produce EAD patterns with decreasing amplitudes which, however, are less observed in practice, see S2 Appendix for details.

## Supporting Information

S1 Appendix Two More EAD Patterns with Growing Amplitudes via Stable Limit Cycles in the AP Fast Subsystem. (PDF)

S2 Appendix Comments on EADs with Decreasing Amplitudes. (PDF)
