# Supplementary material for: Early Afterdepolarizations with Growing Amplitudes via Delayed Subcritical Hopf Bifurcations and Unstable Manifolds of Saddle Foci in Cardiac Action Potential Dynamics
Source: PLoS One. 2016 Mar 15;11(3):e0151178. doi: 10.1371/journal.pone.0151178 (PMC4792449; doi:10.1371/journal.pone.0151178)
Supplement: S1 Appendix — (PDF) [file pone.0151178.s001.pdf]

# S1 Appendix

## Two More EAD Patterns with Growing Amplitudes via Stable Limit Cycles in the Fast AP Subsystem

In our study, we found two additional types of EAD generation with growing amplitudes that can be associated with the bifurcation constellation characterized by the stable limit cycles in the fast subsystem of the action potential model.

**Pronounced Delay Effect** Fig. 1 illustrates the scenario in which the trajectory of the AP system still passes through the supercritical Hopf point but now undergoes a much more pronounced delay effect - in vicinity of the supercritical Hopf bifurcation the oscillations are too small to be visible. Only in some distance from the Hopf point the oscillations become visible again due to a jump into the basin of attraction of the stable limit cycles.

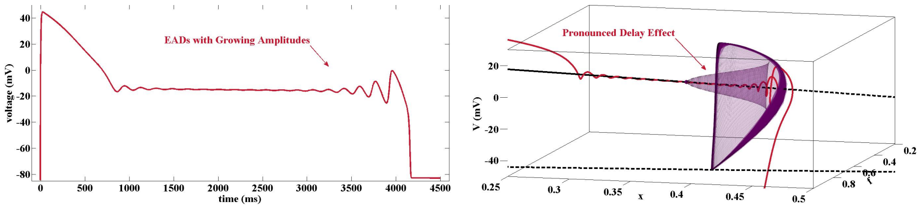


Figure 1: **Generation of EADs with growing amplitudes by passage through a supercritical Hopf bifurcation with pronounced delay effect.** In vicinity of the Hopf point the oscillations are too small to be observed, only in some distance from the Hopf point the trajectory starts to follow the branch of stable limit cycles of the fast subsystem before their extinction at the saddle-homoclinic bifurcation.

**No Passage Through Hopf Point** Second, Fig. 2 illustrates that an actual passage through the supercritical Hopf point is not needed for the generation of EADs with growing amplitudes. In this alternative, the trajectory of the AP system passes by the basin of attraction of the stable focus nodes and is rather directly attracted towards the stable limit cycles from outside.

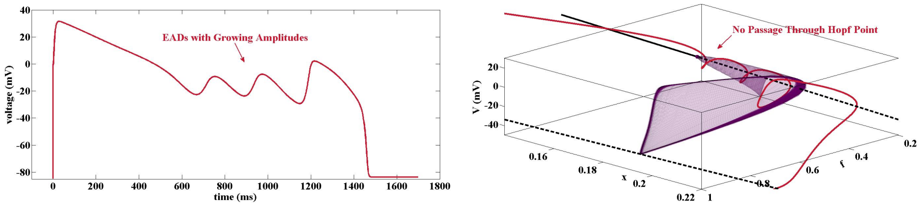


Figure 2: **Generation of EADs with growing amplitudes via direct attraction towards the stable limit cycles from outside.** As opposed to the scenarios of the Tourbillon and the pronounced delay effect, the trajectory does not pass through the supercritical Hopf point. Again, EAD termination takes place via the saddle-homoclinic bifurcation.
