# Supplementary material for: Early Afterdepolarizations with Growing Amplitudes via Delayed Subcritical Hopf Bifurcations and Unstable Manifolds of Saddle Foci in Cardiac Action Potential Dynamics
Source: PLoS One. 2016 Mar 15;11(3):e0151178. doi: 10.1371/journal.pone.0151178 (PMC4792449; doi:10.1371/journal.pone.0151178)
Supplement: S2 Appendix — (PDF) [file pone.0151178.s002.pdf]

S2 Appendix

Comments on EADs with Decreasing Amplitudes

For the sake of completeness we mention that cardiac AP models may also produce EAD patterns with decreasing amplitudes which, however, are less observed in experiments. It is argued in [1], [2] that if a Hopf-homoclinic bifurcation does not exist in the AP fast subsystem, the EAD oscillations (need to) dampen out. This statement was proven incorrect in the main text of our paper (see Section on Unstable Manifold of Saddle Focus). Furthermore, the example given in Fig. 1 shows that EAD oscillations with decreasing amplitudes

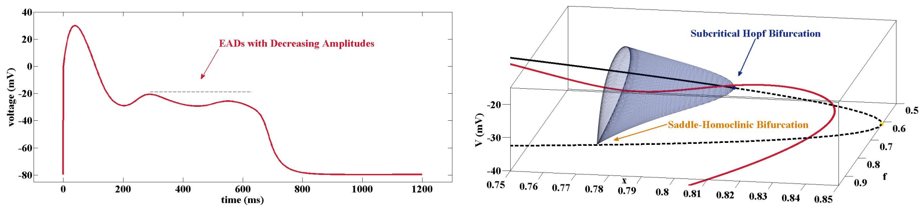

Figure 1: **EADs with decreasing amplitudes in the presence of a Hopf-homoclinic bifurcation.** the trajectory is only affected by the branch of the stable foci with decreasing real parts of the eigenvalues.

may occur even in the presence of a Hopf-homoclinic bifurcation (obtained with the three-dimensional AP model and parameter column B. Here, the Hopf point is basically ignored by the trajectory due to its local direction and speed such that only the branch of the stable foci with decreasing real parts of the eigenvalues has an impact. While the bifurcation properties of the fast subsystem do not depend on the dynamics of the gating variable x, the trajectory of the full system of course does. It is the relation of latter (in combination with the initial conditions) to the fast subsystem that decides if and how the trajectory is affected by the bifurcations. This is further illustrated in Fig. 2 with model

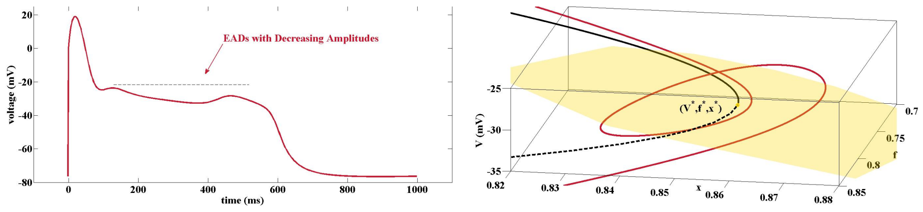

Figure 2: **EADs with decreasing amplitudes in the presence of an unstable manifold of a saddle focus.** The trajectory is repelled from the unstable manifold of the saddle focus (V^*, f^*, x^*) only after a single turn such that an increase of amplitudes is prevented.

parameter column C. As a consequence, the trajectory now only takes a single

turn in the unstable manifold of the saddle focus before rejection, then resulting in an EAD pattern with decreasing amplitudes.

References

[1] Xie Y, Izu LT, Bers DM, Sato D. Arrhythmogenic Transient Dynamics in Cardiac Myocytes. Biophysical Journal. 2014;106(6):1391 – 1397. Available from: `http://www.sciencedirect.com/science/article/pii/S0006349514000848`.

[2] Song Z, Ko CY, Nivala M, Weiss JN, Qu Z. Calcium-Voltage Coupling in the Genesis of Early and Delayed Afterdepolarizations in Cardiac Myocytes. Biophysical Journal. 2015;108(8):1908 – 1921. Available from: `http://www.sciencedirect.com/science/article/pii/S0006349515002441`.
